# Cardiorespiratory fitness, body mass index, and cancer mortality: a cohort study of Japanese men

**DOI:** 10.1186/1471-2458-14-1012

**Published:** 2014-09-27

**Authors:** Susumu S Sawada, I-Min Lee, Hisashi Naito, Ryo Kakigi, Sataro Goto, Masaaki Kanazawa, Takashi Okamoto, Koji Tsukamoto, Takashi Muto, Hiroaki Tanaka, Steven N Blair

**Affiliations:** Department of Health Promotion and Exercise, National Institute of Health and Nutrition, Tokyo, Japan; Department of Epidemiology, Harvard School of Public Health, Boston, MA USA; Division of Preventive Medicine, Department of Medicine, Brigham and Women’s Hospital and Harvard Medical School, Boston, MA USA; Department of Exercise Physiology, School of Health and Sports Science, Juntendo University, Chiba, Japan; Health Promotion Center, Tokyo Gas Co., Ltd, Tokyo, Japan; Department of Public Health, Dokkyo Medical University School of Medicine, Tochigi, Japan; Laboratory of Exercise Physiology, Faculty of Sports and Health Science, Fukuoka University, Fukuoka, Japan; Department of Exercise Science, Arnold School of Public Health, University of South Carolina, Columbia, USA; Department of Epidemiology and Biostatistics, Arnold School of Public Health, University of South Carolina, Columbia, SC USA

**Keywords:** Epidemiology, Maximal oxygen uptake, Physical activity, Exercise, Smoking

## Abstract

**Background:**

The aim of this study is to investigate the independent and joint effects of cardiorespiratory fitness (CRF) and body mass index (BMI) on cancer mortality in a low body mass index population.

**Methods:**

We evaluated CRF and BMI in relation to cancer mortality in 8760 Japanese men. The median BMI was 22.6 kg/m^2^ (IQR: 21.0-24.3). The mean follow-up period was more than 20 years. Hazard ratios and 95% CI were obtained using a Cox proportional hazards model while adjusting for several confounding factors.

**Results:**

Using the 2nd tertile of BMI (21.6-23.6 kg/m^2^) as reference, hazard ratios and 95% CI for the lowest tertile of BMI (18.5-21.5) were 1.26 (0.87–1.81), and 0.92 (0.64–1.34) for the highest tertile (23.7-37.4). Using the lowest tertile of CRF as reference, hazard ratios and 95% CIs for 2nd and highest tertiles of CRF were 0.78 (0.55–1.10) and 0.59 (0.40–0.88). We further calculated hazard ratios according to groups of men cross-tabulated by tertiles of CRF and BMI. Among men in the second tertile of BMI, those belonging to the lowest CRF tertile had a 53% lower risk of cancer mortality compared to those in the lowest CRF tertile (hazard ratio: 0.47, 95% CI: 0.23-0.97). Among those in the highest BMI tertile, the corresponding hazard ratio was 0.54 (0.25-1.17).

**Conclusion:**

These results suggest that high CRF is associated with lower cancer mortality in a Japanese population of men with low average BMI.

## Background

Cancer is one of the main causes of death in the world. The International Agency for Research on Cancer reported that 7.6 million people died from cancer in 2008 [1]. Furthermore, deaths from cancer worldwide are projected to continue to rise, with an estimated 17 million deaths in 2030 [2]. Thus, the prevention of cancer is an important world health issue.

Previous large cohort studies have shown body mass index (BMI) as an independent risk factor for cancer incidence and mortality [3–8]. In addition, several cohort studies in the North American population have reported that an inverse relationship between cardiorespiratory fitness (CRF), an objective marker of physical activity, and cancer incidence or mortality [9–19]. Similarly, we also have observed that Japanese male workers who have high CRF experience a lower risk of cancer mortality [20], even though there are some differences in patterns of cancer mortality in Japanese men compared to North American men [2].

Further, some studies have shown that although high BMI is an independent risk factor of cancer mortality, higher levels of CRF are associated with lower cancer mortality among those with a high BMI [11, 12, 17]. However, these studies comprised North American participants, who have some of the highest BMI levels in the world and are primarily non-Hispanic white individuals. There are no data regarding the joint effects of CRF and BMI on cancer mortality in Asian populations, who have relatively low BMI levels. We hypothesized that higher CRF and moderate BMI would each be associated with lower cancer mortality risk, independent of the other factor, and also that the lowest risk would occur among individuals in the joint group of highest CRF and moderate BMI. Thus, we intend to extend our previous study, which investigated CRF and cancer mortality, the purpose of the current study was to investigate the joint effects of CRF and BMI on cancer mortality among a Japanese male population with low average BMI.

## Methods

### Participants

All participants were employees of the Tokyo Gas company (Tokyo, Japan) that supplies natural gas to the Tokyo area. The number of personnel in the Tokyo Gas company was 12,899 (male: 11,978 and female: 921) at the end of March 1982 when the study was instituted. Most of the workers were sedentary during a typical work day. Female workers were excluded from the present analysis because of the small number. Participants completed a submaximal exercise test, annual health examinations, and self-administered questionnaires regarding their health habits between1982 and 1988. People who were diagnosed as having cancer, cardiovascular disease, diabetes mellitus, gastrointestinal disease, and tuberculosis based on the baseline health examination between 1982 and 1988 (n = 1162) were excluded from the present study to prevent the influence of these diseases and medication use on the assessment of CRF. In addition, the participants were given an interview at the time of their submaximal exercise test between 1982 and 1988 to check for the presence or absence of asthma, liver disease, kidney disease, bone fractures, torsional deformity of the lumbar spine, lower back pain, and neuralgia. If the participants had any one of these conditions, they were also excluded from the CRF test (n = 1777). Finally, to minimize potential bias from undiagnosed cancer that may have led to weight loss, participants were excluded if they had a BMI that was less than 18.5 kg · m^-2^ (n = 279). After these exclusions, the number of participants included in the present study was 8760 men.

This study was approved by the ethics committee of the National Institute of Health and Nutrition of Japan.

### Health examination and fitness test

In Japan, the Industrial Health and Safety Law and related regulations require the employer to conduct annual health examinations of all employees. Employees are required by law to participate. Height and weight were measured on a standard physician’s scale with light clothing but no shoes. BMI was calculated from measured weight and height. Resting blood pressure was obtained with standard auscultatory methods. A questionnaire was administered before the start of the fitness tests to assess the individual’s smoking and alcohol habits. Participants underwent a submaximal exercise test on a cycle ergometer. The exercise test consisted of two or three four-minute, progressively increasing exercise stages. The initial loads for the participants in the 19–29, 30–39, 40–49, and 50–59 years of age brackets were 600 kpm, 525 kpm, 450 kpm, and 375 kpm, respectively. Heart rate was measured using an electrocardiogram monitor (Cardiosuper 2E32, Sanei Sottki Co. Ltd., Tokyo, Japan), and 85% of their age-predicted maximal heart rate (220 minus age in years), was set as the target heart rate. The load was increased by 225 kpm for each stage among all age groups, until heart rates during the course of the exercise reached the target heart rate, or until completion of the third stage. Based on the work rate obtained from the last one minute of the final stage and heart rate obtained from the last 10 seconds, maximal oxygen uptake (VO2max) was estimated using the Åstrand-Ryhming Nomogram [21] and Åstrand’s Nomogram correction factors [22].

### Mortality surveillance

We followed the participants for cancer mortality until June 30, 2003. The mean follow-up period was 20.2 years (range: 15–253 months). We ascertained mortality in two ways. The data for death occurring during employment at the Tokyo Gas company was available from the company (n = 167). For those who died after retirement, death was identified based on a members list kept by the Tokyo Gas Retiree Office (n = 195). Individuals retired from the company have access to a variety of welfare services for a minimal membership fee, paying this membership fee to the secretariat every year. Because the Office collects this annual fee from members every year, the secretariat has contact annually with the member. This allows annual follow-up of every participant. The majority of retirees subscribe to the Tokyo Gas Retiree Office; only 311 people (3.4%) did not. Thus, these 311 had their follow up time censored at the time they retired from Tokyo Gas Company. Additional details of the mortality surveillance have been previously described [20].

### Data analysis

Man-years was calculated from the year of the baseline examination (1982–1988) to the year of death, loss to follow-up, or June 30, 2003. We categorized men into tertiles based on distributions of estimated VO2max according to several age groups (19–29, 30–39, 40–49, and 50–59 yr of age) as we were concerned about residual confounding by age, even with statistical adjustment. We also categorized men into tertiles (18.5-21.5, 21.6-23.6, 23.7-37.4 kg · m^-2^) based on the distribution of BMI. We used Cox proportional hazards models to evaluate the relationship between cancer mortality and CRF as well as BMI, and estimated p for trend across categories of CRF and BMI. Hazard ratios for cancer mortality and 95% confidence intervals were obtained by using the lowest tertile of CRF and second tertile (21.6-23.6 kg · m^-2^) of BMI as reference. The second tertile of BMI was used as the referent group because data from a large study of East Asians showed lowest mortality in this BMI range [23]. We calculated age- and multivariable-adjusted hazard ratios that adjusted for age (continuous variable), systolic blood pressure (continuous variable), cigarette smoking (never-smokers, past smokers, current smokers of 1–19 cigarettes per day, 20 or more cigarettes per day), alcohol intake (none, 1–45 g per day, 46 g or more per day), and BMI (continuous variable) or CRF (continuous variable). These covariates were selected because they are potential confounders of the associations of CRF or BMI with cancer mortality. Because we were concerned about possible residual confounding by smoking, even with statistical adjustment, we examined associations separately for never smokers and smokers (past smokers, current smokers of 1–19 cigarettes per day, 20 or more cigarettes per day). Further, a statistically significant interaction between smoking status and BMI or CRF was also observed. To assess interaction, multiplicative interaction terms (smoking status × BMI or smoking status × CRF) were included in the multivariable models and statistical interaction was evaluated using the Wald test comparing models with or without these interaction terms. We also studied the relationship between joint categories of CRF and BMI with cancer mortality. Hazard ratios and 95% confidence intervals for cancer mortality were obtained by using the joint category of the second tertile of BMI and the lowest tertile of CRF as reference. In the multivariable model, we adjusted for age, systolic blood pressure, cigarette smoking, and alcohol intake. The multiplicative interaction term (BMI × CRF) was included in a model to examine statistical interaction. To test proportional hazards assumptions, we examined log-minus-log plots and no evidence was found of violation of proportionality assumptions. All analyses were performed using SPSS, version 15.0 J for Windows (SPSS, Inc., Chicago, IL). All P values provided are from two-sided tests.

## Results

The number of participants in this study was 8760 at baseline. The median age of participants was 35 yr and the age range was between 19 to 59 yr old. At baseline, the mean (SD) BMI of the study participants was 22.8(2.4) kg · m^-2^. According to BMI classification from the World Health Organization, 55% were normal low range (18.5–22.9 kg · m^-2^), 26% were normal high range (23.0–24.9 kg · m^-2^), 18% were overweight (25.0–29.9 kg · m^-2^) and only one percent was obese (≥30.0 kg · m^-2^).

The mean follow-up time was 20.2 years, and the total follow-up experience for the cohort was 176,736 man-years of observation. There were 345 all-cause deaths and 178 cancer deaths during the follow-up time. This included deaths from lung cancer (47 cases), stomach cancer (34 cases), liver cancer (22 cases), colorectal cancer (18 cases), and esophageal cancer (12 cases); there were 45 deaths from other cancers.

Table 1 shows baseline characteristics of survivors, decedents from any cause and cancer, and men categorized by BMI and CRF levels. Survivors were younger in age at baseline than decedents from any cause or from cancer. Men who died of cancer had a higher prevalence of smoking. Men who were lean tended to have lower blood pressure and higher CRF, but they also had the highest smoking rate compared to the other groups. Similarly, men who were more fit tended to have lower blood pressure and lower BMI.

**Table 1:** **Baseline characteristics of survivors, decedents from any cause and from cancer**

Characteristic	*N*	Age	VO2max	BMI	SBP	DBP	Smokers	Drinkers
		(yr)	(mL · kg ^-1^ · min ^-1^)	(kg · m ^-2^)	(mm Hg)	(mm Hg)	(%)	(%)
Survivors	8,415	34 (28–43)	37.0 ± 6.9	22.8 ± 2.4	124.4 ± 12.1	73.5 ± 10.8	63.4	63.4
Decedents from any cause	345	46 (39–53)	33.7 ± 6.5	22.9 ± 2.7	125.2 ± 12.8	76.8 ± 10.0	68.9	68.9
Decedents from cancer	178	46 (40–53)	33.6 ± 6.9	22.8 ± 2.5	123.3 ± 12.7	75.8 ± 10.5	72.5	72.5
*BMI*								
1st Tertile (18.5-21.5 kg · m^-2^)	2,846	33 (27–41)	39.6 ± 6.8	20.3 ± 0.8	122.6 ± 12.2	70.5 ± 10.6	66.9	66.9
2nd Tertile (21.6-23.6 kg · m^-2^)	2,956	34 (28–43)	37.3 ± 6.7	22.6 ± 0.6	124.1 ± 11.8	73.2 ± 10.6	62.2	62.2
3rd Tertile (23.7-37.4 kg · m^-2^)	2,958	36 (30–44)	33.8 ± 5.9	25.5 ± 1.6	126.4 ± 12.0	77.0 ± 10.0	61.2	61.2
*Cardiorespiratory fitness*								
1st Tertile (Lowest)	2,819	36 (29–44)	30.2 ± 3.4	24.0 ± 2.5	126.8 ± 12.0	76.0 ± 10.3	63.7	63.7
2nd Tertile	3,020	35 (29–43)	36.1 ± 2.9	22.7 ± 2.2	124.2 ± 11.8	73.5 ± 10.7	64.9	64.9
3rd Tertile (Highest)	2,921	33 (27–42)	44.0 ± 5.4	21.9 ± 2.0	122.3 ± 12.1	71.4 ± 10.7	61.6	61.6

VO2max, maximal oxygen uptake BMI, body mass index.

Data represents median (25th-75th percentile), mean ± SD, or %.

Smokers, percentage of current smokers (one or more cigarettes per day).

Drinkers, percentage of current drinkers (1–45 g per day or 46 g or more per day).

Table 2 shows the hazard ratios for cancer mortality by BMI and CRF groups among never-smokers and current smokers. Overall, there was a statistically significant interaction between smoking status and BMI (P for interaction < 0.001). However, there was no interaction between smoking status and CRF (P for interaction = 0.16). For never-smokers, men in the highest tertile of BMI had a high, non-significant hazard ratio compared with the reference group (second tertile). For current smokers, the lowest and highest tertiles of BMI had lower hazard ratios than the reference group. Meanwhile, there was an inverse dose–response trend across CRF levels for cancer mortality. The group with the highest CRF had a 45% lower risk of cancer mortality than the group with the lowest CRF among current smokers (hazard ratio: 0.55, 95% confidence interval: 0.34-0.89). Among never-smokers, the hazard ratios of CRF were unstable, likely due to the small sample size.

**Table 2:** **Hazard ratios for cancer mortality by body mass index and cardiorespiratory fitness among never-smokers and current smokers**

Variable	*N*	Man-years	No. of cancer deaths	Age adjusted hazard ratio	Multivariable hazard ratio
				(95% CI)	(95% CI)
**Never-smokers (n = 2,152)**					
*BMI*					
1st tertile (18.5-21.5 kg · m-2)	716	14,550	11	0.93 (0.39 - 2.18)	0.96 (0.41 - 2.27)
2nd tertile (21.6-23.6 kg · m-2)	725	14,681	10	1.00 (Reference)	1.00 (Reference)^a^
3rd tertile (23.7-37.4 kg · m-2)	711	14,483	13	1.07 (0.47 - 2.44)	1.23 (0.51 - 2.23)
*P for trend*				0.727	0.576
*Cardiorespiratory fitness*					
1st tertile (Lowest)	704	14,253	13	1.00 (Reference)	1.00 (Reference)^b^
2nd tertile	760	15,555	9	0.68 (0.29 - 1.58)	0.67 (0.28 - 1.60)
3rd tertile (Highest)	688	13,905	12	1.02 (0.46 - 2.23)	0.99 (0.43 - 2.28)
*P for trend*				0.990	0.986
**Current smokers (n = 5,552)**					
*BMI*					
1st tertile (18.5-21.5 kg · m-2)	1,766	35,646	36	0.79 (0.52 - 1.22)	0.73 (0.47 - 1.13)
2nd tertile (21.6-23.6 kg · m-2)	1,903	38,110	49	1.00 (Reference)	1.00 (Reference)^a^
3rd tertile (23.7-37.4 kg · m-2)	1,883	37,902	44	0.84 (0.56 - 1.26)	0.72 (0.47 - 1.10)
P for trend				0.856	0.923
*Cardiorespiratory fitness*					
1st tertile (Lowest)	1,794	35,798	51	1.00 (Reference)	1.00 (Reference)^b^
2nd tertile	1,960	39,610	50	0.88 (0.60 - 1.30)	0.82 (0.55 - 1.23)
3rd tertile (Highest)	1,798	36,250	28	0.60 (0.38 - 0.95)	0.55 (0.34 - 0.89)
P for trend				0.032	0.015

BMI, body mass index.

^a^ Adjusted for age, cardiorespiratory fitness, systolic blood pressure, and alcohol intake.

^b^ Adjusted for age, BMI, systolic blood pressure, and alcohol intake.

We calculated age and multivariable-adjusted hazard risks for cancer mortality by cross-tabulating tertiles of CRF and tertiles of BMI (Table 3). Men in the lowest tertile of CRF and the second tertile of BMI were used as reference. Among those in the second tertile of BMI (21.6-23.6 kg/m2), those in the highest CRF tertile had a 55% lower age adjusted hazard ratio of cancer mortality compared to those in the lowest CRF tertile. After additional adjustment for systolic blood pressure, smoking status, and alcohol intake, the results were attenuated but not substantially changed (hazard ratio: 0.47, 95% confidence interval: 0.23-0.97).

**Table 3:** **Hazard ratios for cancer mortality according to combined body mass index and cardiorespiratory fitness groups**

Variable	*N*	Man-years	No. of cancer deaths	Age adjusted hazard ratio	Multivariable hazard ratio
				(95% CI)	(95% CI)
1st tertile of BMI (18.5-21.5 kg · m^-2^)					
Low fitness	484	9,578	15	1.18 (0.62 - 2.26)	1.13 (0.59 - 2.17)
Moderate fitness	996	20,029	25	0.97 (0.55 - 1.70)	0.90 (0.51 - 1.58)
High fitness	1,366	27,505	22	0.73 (0.40 - 1.30)	0.70 (0.39 - 1.26)
*P for trend*				0.141	0.145
2nd tertile of BMI (21.6-23.6 kg · m^-2^)					
Low fitness	824	16,606	23	1.00 (Reference)	1.00 (Reference)^a^
Moderate fitness	1,117	22,734	22	0.72 (0.40 - 1.30)	0.70 (0.39 - 1.27)
High fitness	1,015	20,531	11	0.45 (0.22 - 0.93)	0.47 (0.23 - 0.97)
*P for trend*				0.027	0.037
3rd tertile of BMI (23.7-37.4 kg · m^-2^)					
Low fitness	1,511	30,304	34	0.81 (0.48 - 1.37)	0.82 (0.48 - 1.39)
Moderate fitness	907	18,469	17	0.66 (0.35 - 1.24)	0.66 (0.36 - 1.24)
High fitness	540	10,979	9	0.54 (0.25 - 1.17)	0.54 (0.25 - 1.17)
*P for trend*				0.254	0.267

BMI, body mass index.

Low fitness: 1st tertile of fitness, Moderate fitness: 2nd tertile of fitness, High fitness: 3rd tertile of fitness.

^a^Adjusted for age, systolic blood pressure, cigarette smoking, and alcohol intake.

There was no statistically significant interaction between BMI and CRF (P for interaction = 0.73). Inverse dose response trends across fitness levels for cancer mortality were observed in all BMI tertiles, with the trend being statistically in the 2nd BMI tertile but not the 1st and 3rd tertile BMI tertiles likely because of limited statistical power.

Pre-existing cancer at baseline, as yet undiagnosed, may decrease CRF and/or BMI and lead to biased results. To address this, we examined the hazard ratios for cancer mortality by CRF and BMI groups according to follow-up period (Table 4). The findings for follow-up ≥10 years were, compared with shorter follow-up periods, but CRF continued to have significant inverse associations with cancer mortality even in the group with longer follow-up. The stronger association in the first 10 years of follow-up may have been due to undiagnosed cancer at baseline and/or changes in CRF during follow-up.

**Figure 1:**
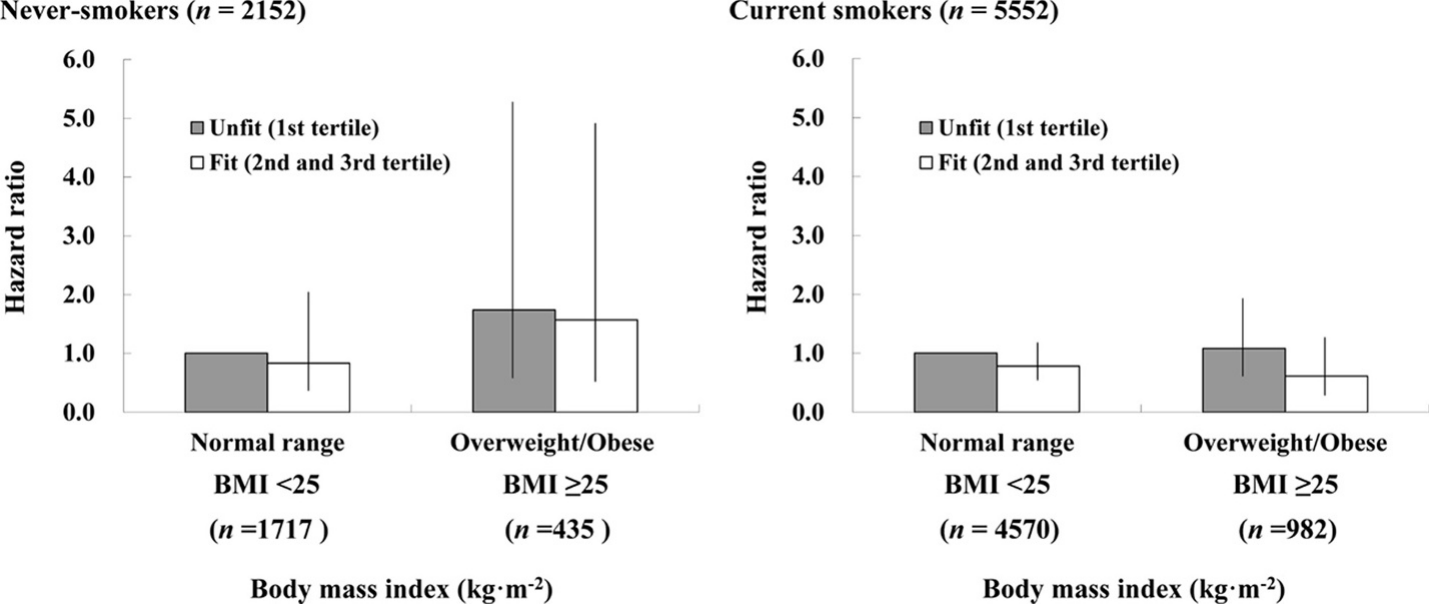
**Joint effects of cardiorespiratory fitness and body mass index on cancer mortality among never-smokers and current smokers.** Adjusted for age, systolic blood pressure, alcohol intake. Each bar shows 95% confidence interval.

A further concern is residual confounding by smoking, even with statistical adjustment. Thus, we examined associations separately for never smokers and smokers. Figure 1 shows the multivariable-adjusted hazard ratios for cancer mortality by joint CRF and BMI groups among never-smokers and smokers. Because of the small numbers of cancer deaths, the second and highest tertiles of CRF were combined into one group (fit). Similarly, BMI were divided into two groups according to BMI classification from the World Health Organization, normal (BMI <25) versus overweight/obese (BMI ≥25). For never-smokers, the highest cancer mortality rate occurred among unfit men in the overweight/obese group; however, for smokers, the highest cancer mortality rate was among unfit men in the normal BMI group, likely reflecting bias from smoking-related illness and leanness. Among both never-smokers and smokers, fit men had lower cancer mortality rates than unfit men in all BMI groups.

**Table 4:** **Hazard ratios for cancer mortality by body mass index and cardiorespiratory fitness at baseline, according to follow-up period**

Variable	N	Man-years	No. of cancer deaths	Age adjusted hazard ratio	Multivariable hazard ratio
				(95%CI)	(95%CI)
< 10 yr of follow-up (n = 8,760)					
*BMI*					
1st tertile (18.5-21.5 kg · m^-2^)	2,846	29,323	12	1.47 (0.62 - 3.49)	1.50 (0.50 - 4.51)
2nd tertile (21.6-23.6 kg · m^-2^)	2,956	28,162	9	1.00 (Reference)	1.00 (Reference)^a^
3rd tertile (23.7-37.4 kg · m^-2^)	2,958	29,342	14	1.43 (0.62 - 3.30)	1.33 (0.41 - 4.36)
*P for trend*				0.983	0.598
*Cardiorespiratory fitness*					
1st tertile (Lowest)	2,819	27,905	18	1.00 (Reference)	1.00 (Reference)^b^
2nd tertile	3,020	29,959	11	0.59 (0.28 - 1.26)	0.55 (0.25 - 1.18)
3rd tertile (Highest)	2,921	28,962	6	0.37 (0.15 - 0.93)	0.35 (0.13 - 0.92)
P for trend				0.026	0.024
≥ 10 yr of follow-up (n = 8,523)					
*BMI*					
1st tertile (18.5-21.5 kg · m^-2^)	2,883	56,531	50	1.21 (0.81 - 1.80)	1.20 (0.80 - 1.79)
2nd tertile (21.6-23.6 kg · m^-2^)	2,758	59,378	47	1.00 (Reference)	1.00 (Reference)^a^
3rd tertile (23.7-37.4 kg · m^-2^)	2,882	59,231	46	0.90 (0.60 - 1.35)	0.86 (0.57 - 1.30)
*P for trend*				0.154	0.123
*Cardiorespiratory fitness*					
1st tertile (Lowest)	2,733	55,914	54	1.00 (Reference)	1.00 (Reference)^b^
2nd tertile	2,943	60,703	53	0.93 (0.63 - 1.35)	0.86 (0.58 - 1.26)
3rd tertile (Highest)	2,847	58,522	36	0.72 (0.47 - 1.10)	0.67 (0.43 - 1.04)
P for trend				0.139	0.077

BMI, body mass index.

^a^ Adjusted for age, cardiorespiratory fitness, systolic blood pressure, cigarette smoking, and alcohol intake.

^b^ Adjusted for age, BMI, systolic blood pressure, cigarette smoking, and alcohol intake.

## Discussion

We investigated the independent and joint effects of CRF and BMI on cancer mortality in a Japanese population with low average BMI. In addition, we examined the joint effects by smoking status and follow-up period to minimize bias from pre-existing, undiagnosed cancer at baseline. The main finding was that men who had high CRF had a lower risk of cancer mortality, regardless of their BMI.

### Body mass index and cancer mortality

Previous large cohort studies have shown that high BMI is a risk factor for all cancer mortality, as well as site-specific cancer mortality [4–8]. Calle et al. investigated the relationship between BMI and cancer mortality among approximately 900,000 American people [5]. The results of their study showed that high BMI is a risk factor for overall cancer mortality, but they did not have measures of CRF for adjustment. Recently, Zheng et al. investigated the relation between BMI and mortality in a pooled analysis of more then 1.1 million Asians [23]. They reported a J-shaped association between BMI and the risk of cancer death in East Asians. Our results showed no clear relationship between BMI and cancer mortality. These results may reflect the narrow range of BMI among participants in our study.

### Fitness and cancer mortality

Previous analyses from the Aerobics Center Longitudinal Study (ACLS) have examined the relationship between CRF and cancer mortality in a North American population. These studies showed inverse relationships between CRF and cancer incidence or mortality [9, 12, 13, 19]; smoking related cancer [14]; and digestive [17], breast [16], prostate [10, 15], and lung cancer [18]. In addition, Laukkanen et al. also reported an inverse relationship between CRF and cancer mortality among Finnish men [24]. We previously reported, using the same cohort, that Japanese male workers with high CRF had lower cancer mortality rates [20], consistent with the American and Finnish data. The present study extends these findings by examining cancer mortality rates among men jointly classified by BMI and CRF.

### Fitness, body mass index, and cancer mortality

Two papers from the ACLS have published the combined effects of CRF and BMI on cancer mortality. Farrell et al. investigated the joint effects of CRF and several adiposity factors, such as BMI, percent body fat, and waist circumference, on cancer mortality among men [12]. Peel et al. also investigated the joint effects on mortality from digestive cancer [17]. Data from another study, the Lipid Research Clinics Study (LRCS), were used to investigated the joint effects of CRF and BMI on total cancer ( apart from skin cancer) mortality [11]. These studies showed that men with higher CRF have lower cancer mortality rates compared to men having lower CRF, regardless of BMI levels. Participants of the ACLS and LRCS were from a North American population and their average BMI was approximately 26 kg · m^-2^. It is unclear whether these findings are applicable to other populations where the average BMI may be lower, such as in Asian populations. Therefore, this study extends the results of previous investigations to a Japanese population, where the average BMI is low.

### Possible mechanisms

The biological mechanisms for the joint effect of CRF and BMI on cancer mortality are not precisely known. However, several mechanisms may explain why men who have high CRF are protected against cancer mortality, even in a lean population.

First, excess secretion of insulin and insulin-like growth factor, a cancer cell promoter [25], may be inhibited as a result of improved insulin sensitivity resulting from physical activity [26]. An increase in GLUT4 has been attributed to physical activity, with the possible effect of enhancing insulin sensitivity [27]. It also has been reported that muscle contraction itself may be a stimulus for glucose intake into skeletal muscles [28], which then suggests the possibility that physical activity may correct hyperglycemia, thereby preventing cancer cell proliferation. Second, cancer cell promotion may be inhibited by immune functions enhanced by physical activity [26]. Moreover, physical activity increases skeletal muscle mass [29]. Muscle mass conserves large amounts of protein, which plays an important role in the functioning of the immune system [30]. A major cause of cancer is increased and/or chronic inflammation that stimulates cell proliferation. Physical activity reduces adipocyte infiltration into tissues that generates adipokines such as tumor necrosis factor and interleukin-6 that increase inflammation [31]. Thus, systemic effects of these cytokines and hormones may contribute to reduced risk of cancer in physically fit people. Third, aerobic exercise may inhibit DNA oxidation and prevent cancer cell initiation [32, 33], despite the increase in free radicals that accompanies an increase in energy consumption caused by physical activity.

### Strengths

The strength of the present study is the use of a unique population with a low average BMI. Previous reports on this issue have investigated North American populations who have relatively high BMI. We measured CRF, a more objective measure compared with self-reported physical activity [34]. Although, our measurements were indirect, Teräslinna et al. investigated the relationship between measured VO2max and estimated VO2max using the Åstrand-Ryhming Nomogram and correction factors utilized in the present study, obtaining a correlation coefficient of 0.92 [35].

### Limitations

There also are several limitations in this study. First, because of small number of cancer deaths, we could not analyze site-specific cancers. Cancer is a collection of heterogeneous diseases with different aetiological pathways. Thus, site-specific cancer analyses are needed in future. In addition, low statistical power may lead to non-statistically significant findings. Future studies with large sample size are needed to confirm the present findings. Second, cause of death was reported in an interview with the next of kin, leading to a possible misclassification of the cause of death. However, it is assumed that in most cases, attending physicians directly informed the next of kin of the cause of death. Therefore, this is unlikely to be a major limitation. Third, certain dietary factors, such as an anti-oxidants, dietary fiber, omega-3 fatty acids, etc. may reduce cancer incidence [36]. We did not have information on diet and so residual confounding by diet may be present. Finally, we included Japanese men: it is unclear how these findings may apply to other Asian populations who have relatively low BMI levels in general. Furthermore, participants were employees of a single, Japanese urban company and may not be representative of other Japanese. This limits the generalizability of this study but not its validity.

## Conclusions

The present study suggests that high CRF may reduce cancer mortality rates, regardless of BMI levels, in a Japanese population of men with low average BMI. Hence, even populations with lean BMI should be encouraged to be physically active and increase or maintain high CRF to reduce their risk of cancer mortality.
